# Maternal Perceptions of Self-Weight and Child Weight May Influence Milk Choice of Participants in the Special Supplemental Nutrition Program for Women, Infants, and Children (WIC)

**DOI:** 10.1155/2019/3654728

**Published:** 2019-01-03

**Authors:** Loan P. Kim, Nelly Mallo

**Affiliations:** ^1^Pepperdine University, Malibu, CA, USA; ^2^PHFE-WIC, Irwindale, CA, USA

## Abstract

**Objective:**

To examine the association between maternal perceptions of self-weight and child weight status and milk consumption behavior of Hispanic WIC participants.

**Methods:**

Mixed methods design with phone survey (*n*=529) and one-on-one interviews (*n*=35). Demographic characteristics, Chi-square, and thematic analyses were conducted.

**Results:**

More than half of overweight mothers misperceived their own weight status as well as those of their children. Mothers who perceived their child to be overweight were more likely to make a healthier food choice for their family, namely, choosing reduced-fat milk instead of whole milk. Qualitative interviews revealed a cultural preference for larger size children, and mothers defined healthy weight for their child as a function of (1) the child's ability to be as active as other children their age, regardless of child's BMI, and (2) the pediatrician's opinion of the child's weight status.

**Conclusions:**

Maternal perception of self-weight and child weight status seems to guide milk choices. Mothers may have some level of recognition of overweight in themselves and their child, thus adopting healthier milk choices. Culturally related perceptions should be considered when designing obesity prevention strategies, and the role of a pediatrician cannot be understated when developing obesity prevention programs for Hispanic families.

## 1. Introduction

Obesity rates have reached epidemic proportion in the United States among adults, with similar trends seen in children. Overweight in children is defined as a body mass index (BMI) at or above the 85^th^ percentile but lower than the 95^th^ percentile, and obesity is defined as equal to or above the 95^th^ percentile BMI for age and sex based on the Centers for Disease Control and Prevention (CDC) reference data [1]. Long-term health consequences of obesity include diabetes, heart disease, several types of cancers, cardiovascular disease, and premature death [2, 3]. Furthermore, overweight children are more likely to become severely overweight as adults, with significant chronic disease implications [4]. Recent data from 2012 found that more than one-third of children and adolescents were overweight or obese [5]. The prevalence of obesity among children aged 2 to 5 years has decreased from 13.9% in 2003-2004 to 8.4% in 2011-2012 [1], and this decline is controversial [1, 6].

Hispanics represent the largest immigrant group in the US, but they bear a disproportionately higher burden of obesity; NHANES data from 2011 to 2012 reveal significant disparities in obesity rates among low-income and ethnic minority children; the prevalence among children and adolescents was higher among Hispanics (22.4%) than non-Hispanic blacks (20.2%) and non-Hispanic whites (14.1%) [7]. A cross-sectional study of preschool-aged children in 20 urban cities in the US found that Hispanic children had the highest prevalence of obesity (25.8%) compared to non-Hispanic white children (14.8%) [8]. Children aged 2–5 of Hispanic descent are more likely to be overweight than their non-Hispanic white or black counterparts [5, 9].

As primary caregivers, mothers are most influential in modeling dietary behavior for their children [8, 9]. Since dietary behaviors develop and solidify in early childhood, understanding how mothers' perceptions of their own weight and that of their children impact child feeding practices are important issues to explore. The literature on maternal perception of their child's weight status suggests that parents generally fail to correctly identify obesity in their young child [10–13]. Using nationally representative NHANES III data in 2001, Maynard et al. examined maternal perception of overweight among 5500 children aged 2–11 years and reported that nearly a third of mothers identified their overweight child as “about the right weight” [14]. A systematic review conducted by Rietmeijer-Mentink and colleagues to examine the difference between paternal perception and actual weight status of children found that about two-thirds of overweight children were incorrectly perceived as having normal weight by their parents; more importantly, it was noted that this misperception of overweight status in children was higher among parents with children aged 2–6 years old [15]. Francescatto and colleagues also reviewed the literature on maternal perceptions of the nutritional status of their overweight children; these researchers found that the proportion of mothers who inaccurately perceived the nutritional status of their children was high, and the more common underestimation was among those with an overweight or obese child [16]. These findings suggest the importance of accurate maternal perception as a first step toward successful obesity prevention efforts in young children.

While a majority of the literature on maternal perception has focused on quantifying and determining predictors of accurate child weight status [17, 18], there is a small but growing body of qualitative studies which have explored how cultural factors impact maternal perception of child's weight status; many of these studies have focused on Hispanic mothers and their children. Lindsay and colleagues reported a cultural preference for a “chubby child” among Hispanic families as this suggested the child was robust and healthy [19]. Other researchers have documented that Hispanic mothers and children define overweight as a function of declining physical abilities, such as tiring easily, difficulty breathing during an activity, or not being able to keep up with other kids, and not a function of actual weight or BMI status [20, 21].

The Special Supplemental Nutrition Program for Women, Infants and Children (WIC) is a food and nutrition education program for pregnant, breastfeeding, and postpartum women, infants, and children under age five who are low income (up to 185% of the Federal Poverty Level) and at nutritional risk. Nationwide, approximately 25% of the individuals served are women, and approximately half of these women are pregnant while the other half are postpartum. WIC services are available in every state and US territory, and currently WIC services are delivered to over 8 million participants each month [22]. In 2016, in Los Angeles County (LAC), WIC served close to half a million individuals in the county, which includes 326, 167 children under age 5, accounting for approximately 50% of all children under age 5 in Los Angeles County [23]. Because WIC is uniquely positioned to effect health outcomes of ethnic minority and underresourced families, research to better understand maternal perceptions of child weight status in this population is needed. To date, there is a critical gap in the literature, with few studies on maternal perceptions among WIC mothers. In a small sample of WIC participants in Kentucky, Baughcum et al. reported 79% of mothers of overweight children aged 2–5 years did not perceive their child to be overweight [10]. Our own work with WIC participants in California found that more than 75% of all mothers in the study classified their overweight or obese child as being “about the right weight.” Additionally, our study found that higher maternal BMI was associated with less accurate maternal perception of child weight [17].

While a growing number of studies have documented inaccurate perceptions of child weight or how feeding styles were associated with child weight status [24–29], we are not aware of any studies that have examined the association between parental perceptions and actual feeding practices of preschool-age children. This study seeks to determine whether there is a link between maternal perceptions of self-weight and child's weight status and the type of milk a mother chooses for herself and her child. Determining the relationship between maternal perception of a child's weight and actual feeding behavior can be vital for programming WIC services to counsel mothers about their child's weight.

In this study, we focused on milk as the primary feeding practice for the following reasons. First, milk is a significant component of the WIC food package for children aged 2–5 and is an important source of calories and nutrients for preschool-aged children. The WIC program provides a supplemental food package to about 50% of all infants and 25% of all children in the US under age 5 [30]. When this study was conducted in 2007–2009, parents had the option of purchasing any type of milk (whole, reduced fat, or nonfat) with their WIC vouchers. Second, research documents that whole milk contributes the most significant source of saturated fat, particularly in diets of low-income Hispanic children [31, 32]. Third, it has been noted in the literature that there is a cultural preference in the Hispanic population to favor whole milk over lower fat options [33, 34]. Overall, we focused on milk because, in the early childhood years, milk is one of the most commonly consumed foods and one that presents options for parents, from less healthy whole and flavored milk to more healthy lower fat options. Additionally, the analysis of milk (whole versus reduced fat) is grounded on the recommendations by the American Academy of Pediatrics and the American Heart Association to provide reduced-fat milk for young children as the healthier option over whole milk.

## 2. Methods

This study employed mixed methods and utilized secondary data analysis of the Children's Health Information Research Project (CHIRP) study, with the addition of one-on-one qualitative interviews to provide a holistic understanding of how maternal perceptions of weight status impact feeding practices.

### 2.1. Quantitative Survey (CHIRP)

At the time of the study in 2007–2010, the CDC definition of overweight was defined as at or above the 95th percentile BMI for age and sex and “at risk of overweight” as between the 85th to 95th percentile of BMI. Current CDC definition has shifted the “at risk of overweight” to “overweight”; the “overweight” categorization is now “obese” [35]. Henceforth, we apply the current CDC definition in this study. The Children's Health Information Research Project (CHIRP) was a case-control study which examined the relative impact (if any) of total family exposure to the WIC program on the risk of childhood obesity among three- and four-year-old children in Los Angeles County. More details of the study design are described elsewhere [36]. Over a 14-month period in 2007-2008, participants were drawn from a single large WIC site east of Los Angeles. Mothers of the first presenting child aged 36–59 months who met the obesity criterion (>95th percentile) were invited to participate, and if she agreed and enrolled in the study, then the mother of the next presenting child of the appropriate age but within the normal weight range (>5^th^ percentile but <85^th^ percentile) was invited. After informed consent, height and weight measurements were obtained in person for the mother and child at the WIC center. WIC staff followed the protocol for obtaining height and weight measurements; this method has been previously validated [37]. An appointment was made for the phone interview. Exclusion criteria included children who were underweight (<5^th^ percentile) and overweight (85^th^–94.9^th^ percentile).

Survey interviewers were bilingual WIC paraprofessional staff who were blinded to the weight status of the child with whose mother they were speaking with on the phone. The telephone interview was conducted in English or Spanish, based on the participant's preference, and lasted an average of 60 minutes. Questions that addressed perception of maternal and child weight included, “Currently, would you describe yourself/your child as overweight, normal weight or thin?” To assess milk intake, mothers were asked, “What kind of milk do you/your child drink?,” and response options included the following: do not drink milk, whole, reduced fat (1 or 2%), nonfat, chocolate, soy, and rice milk. All data were entered in a bilingual computer-assisted system during the interview and then merged and cleaned for analyses. The study was approved by the Institutional Review Boards at the University of California, Los Angeles (UCLA), and Independent Review Consulting, Inc.

To determine the concordance between objectively defined weight status and maternal perception of self-weight and child weight status, we compared anthropometrically derived mother and child BMIs against the subjective perception question, “Currently, would you describe yourself/your child as overweight, normal weight or thin?” Maternal education was categorized as less than 12 years, high school graduate, college, or more. Birthplace was dichotomized as either US born or foreign born. The milk variable was dichotomized as whole or reduced fat (2%, 1%, and nonfat milk). Other milk types (soy, rice, and chocolate) had small frequencies in each category and subsequently grouped together in the analyses.

### 2.2. Statistical Analysis

Descriptive statistics was conducted using SPSS 23 (Chicago, IL) to describe the demographic characteristics of the sample, and BMI weight status of mother and child was computed. Pearson's chi-square analyses were performed to compare associations between obese and normal weight children and mothers with regard to their perception of weight status and milk intake, and statistical significance was measured at *P* < 0.05. Cohen's Kappa values were calculated to assess inter-rater reliability. Altogether, the study contained a sample size of 655 participants. Of this total, we selected for only Hispanic mother and child dyads, resulting in a sample size of 529 participants.

### 2.3. Qualitative Interviews

In addition to the CHIRP survey data, qualitative one-on-one interviews were conducted to provide a more nuanced understanding of how perception impacts maternal food choice, particularly milk. Following the CHIRP survey, over a 12-month period between 2009 and 2010, one-on-one interviews were conducted with WIC participants and clinic staff. Using purposeful sampling to match participants in the CHIRP survey, mothers were recruited to participate because they are the primary caregivers responsible for food purchase and meal preparation for the family. Inclusion criteria included being a WIC participant over the age of 18 and who self-identified as Hispanic. PHFE-WIC staff recruited a total of 47 participants; of that, 24 expressed interest in participating while the other 23 participants who were recruited declined because of lack of interest or lack of time. Of the 24 recruited, 20 met the criteria and consented to participate. Additionally, we interviewed 15 WIC staff who self-identified as Hispanic. A total of 35 interviews were conducted, 20 with WIC mothers and 15 with WIC staff.

All mothers were recruited from one local PHFE-WIC clinic; front desk staff were recruited from among those who met the criteria, and a bilingual, bicultural research staff contacted each eligible mother for the interview at the next WIC appointment. Interviews were conducted in either English or Spanish, based on the participant's preference; staff interviews were conducted in English after work hours at WIC sites around Los Angeles. Interviews were conducted by researchers (LPK and NM) trained in qualitative research methodology, and each interview lasted approximately one hour. All Spanish-speaking interviews were conducted by NM. All participants received a $30 gift card as compensation for their time. The study was reviewed and approved by the UCLA Institutional Review Board.

Recruitment and consent materials along with interview guides were developed by the research team, with feedback from WIC research staff. A semistructured interview guide with open-ended questions was designed to stimulate discussion about perceptions of self-weight and child's weight, with additional probe questions to clarify participants' responses and focus the discussion. After feedback and discussion with WIC research staff, the interview guide was finalized and translated into Spanish. Translated materials were back translated and pilot tested to ensure integrity and cross-cultural consistency. All interviews were digitally recorded and transcribed verbatim; those conducted in Spanish were first transcribed verbatim in Spanish and later translated into English. An independent bilingual contractor verified the accuracy of the translations.

### 2.4. Qualitative Data Analysis

**Figure 1:**
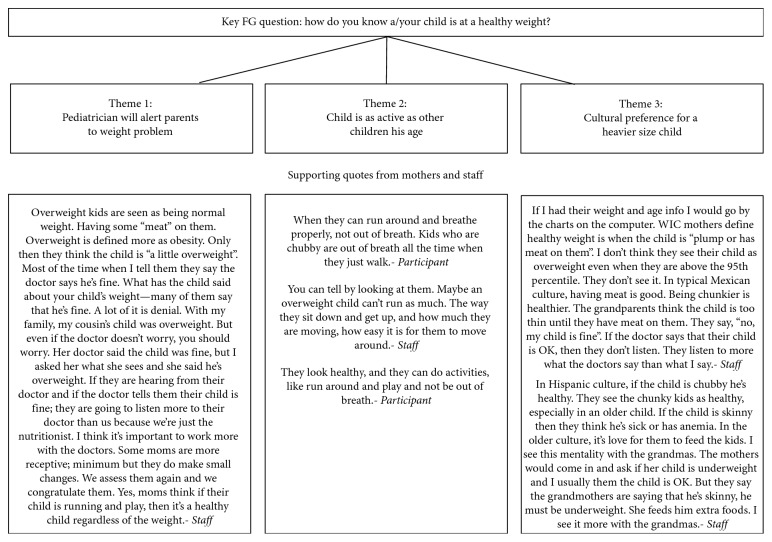
Overview of focus group topics and emerging themes.

Transcripts and interviewer notes were organized and coded in ATLAS.ti software (v.6.1.1., Eden Prairie, MN) and analyzed using thematic analysis [38]. This process involved multiple independent readings of the transcripts by the research team to allow for themes to emerge. After this initial process, researchers (LPK and GGH) convened to discuss the major themes and develop consensus, with NM providing feedback on themes and reconciling differences. Subsequently, a second pass of the transcripts was completed to ensure that all themes were captured. A third reading allowed for any additional themes to be added. Themes were then coded and grouped to form 3 major emerging themes around maternal perceptions of healthy weight. These identified themes were organized and are summarized in Figure 1 using selected quotes from interviews which reflected those discussed with most frequency.

## 3. Results

Table 1 provides data on characteristics of the target child and mother in the CHIRP study. Altogether 529 Hispanic children and their mothers participated, with more than 90% of Mexican descent. Mothers were on average 30.6 years old, and more than half of the mothers had less than a high school education. A majority of the surveys were conducted in Spanish. There was no difference between the two groups with regard to maternal age and birthplace. Average maternal BMI met the criterion for obesity, or nearly so, for both groups but was significantly higher for mothers of obese children. About one-third of all mothers were overweight (BMI ≥ 25), and half were obese (BMI ≥ 30).

Qualitative interview participants were on average 28 years old and had been enrolled in WIC for about 2.3 years. All participants self-identified as Mexican-American; forty-five percent of participant interviews were conducted in Spanish, and the rest were conducted in English. Staff age ranged from 22 years to 33 years (with an average age of 29 years). All staff also self-identified as Mexican-American, and on average, staff were employed at WIC for an average of 8 years.

### 3.1. Maternal Perception of Self-Weight and Child's Weight Status

Results from our analyses suggest that mothers misperceived their own weight status as well as that of their child; mothers more accurately perceived their own weight status than their child's weight status. Overall, 73% of mothers accurately perceived their own weight status, but more than a quarter of participants misperceived their own weight status (Table 2). Cohen's kappa (*κ*) indicated moderate agreement between the mothers' actual weight status compared to their perceived weight status (*κ* = 0.433, *p* < 0.001). This suggests mothers were only moderately reliable in accurately classifying their weight status given their actual weight status. Additionally, to investigate whether mothers accurately perceived their weight status, a chi-square (*χ*^2^) statistic was conducted, and results indicate that among the mothers who were obese, 26% misperceived their weight status and underestimated their weight as “normal” weight (*χ*^2^ = 140.8, df = 1, *N* = 529, *p* < 0.001).

When it came to perceiving their child's weight status, a higher percentage of mothers misperceived their child's weight status. Overall, 63% of mothers accurately perceived their child's weight status, but 36% inaccurately perceived their child's weight status. Among children who were obese (with BMI greater than the 95^th^ percentile), 36% were misperceived by their mothers to be “normal weight” (Table 3). Cohen's kappa (*κ*) found only fair agreement between mothers' perception and their child's actual weight status (*κ* = .224, *p* < 0.001), suggesting that compared to their own weight status mothers were less consistent and thus less accurate, in correctly perceiving their child's weight status compared to their own weight status. More of these mothers actually underestimated their child's weight, perceiving an obese child to be normal weight.

Our findings are supported by other research which found that mothers more accurately perceived their own weight status than that of their child's weight status [39]. Results from qualitative interviews with participants and staff suggest a possible explanation: a cultural preference for larger size children. This cultural preference for a heavier body size for their children has been confirmed in another study with Mexican-Americans [40]. As one staff member stated, “For Mexican-Americans, a big chubby baby is cute and grandmothers love it. They love pinching them, there's a lot to love. They don't see it as overweight. That child eats well and is healthy, that is how they see it.” Additionally, interview data revealed that mothers defined healthy weight for their child as a function of the child's ability to be as active as other children their age, regardless of their BMI percentiles. Second, mothers based their concern about the child's weight on what the pediatrician said about their child (Figure 1). In the Hispanic culture, doctors are held in high esteem; in the following quote, a mother concluded that since the pediatrician showed no concern about her child's weight, there was no need to worry about her child's weight status. The mother reported, “I take him to the doctors where they are more knowledgeable and know which weight he should be [at]. They will tell me if there is a problem with his weight.” These qualitative findings suggest that Hispanic mothers' cultural definition of healthy weight did not always align with the biomedical classification of weight status based on BMI percentiles [13, 40].

### 3.2. Impact of Maternal Perception on Milk Intake

Table 3 summarizes milk choice based on perception of weight status for both mother and child. Overall, about two-thirds of mothers and their children reported drinking reduced-fat milk. Among mothers who perceived their own weight status as overweight, 68% reported choosing reduced-fat milk compared 74% who perceived their weight to be thin and 56% who perceived their weight to be normal. If mothers perceived their own weight status as thin or normal weight, they were more likely to report drinking whole milk. Likewise, among mothers who perceived their child's weight status as overweight, 76% of these children were reported to drink reduced-fat milk compared to 64% overall (*χ*^2^ = 7.9, df = 1, *N* = 529, *p* < 0.05). When we compared anthropometric weight measurements with milk choices, we also found that overweight/obese mothers and obese children were more likely to drink reduced-fat milk than their normal weight counterparts, who were more likely to drink whole milk. Among mothers who were overweight/obese, 30% reported drinking whole milk compared to 44% of mothers who were thin. Also, among children who were obese, only 28% drank whole milk compared to 42% of children who were normal weight.

These data suggest some level of recognition of obesity, and thus healthier choices were made, despite findings from the qualitative interviews which found a strong cultural preference for whole milk. In Spanish, whole milk is *leche entera*, which means “complete milk.” Mothers reported, and staff interviews confirmed, that whole milk is thought to be “complete” because it is “thick” and full of nutrients. While the narratives suggest an overall cultural preference among this sample for whole milk, perceptions of weight status for self and child seemed to affect feeding behavior with regard to milk choice between whole and reduced-fat milk options.

## 4. Discussion

Results of this study support findings in the existing literature on the substantial dissonance between maternal perception and objective biomedical measures of child weight status and thus enhance previous studies through the inclusion of a primarily Hispanic sample and the addition of qualitative methods [12, 14, 17, 18]. This study extends our previous work by examining the association between maternal perception of child weight status and actual feeding choices, in this case milk. Results suggest that while mothers did not accurately assess their child's weight status, they were making feeding choices that suggest they have some recognition of their own and child's weight status. Mothers of an obese or perceived-to-be obese child were more likely to report that the child was drinking reduced-fat milk. The data also suggest that when mothers were actually overweight/obese or perceived themselves to be overweight/obese, they also modified their milk consumption pattern and chose reduced-fat milk. Thus, despite maternal misperception of child's weight status, our findings suggest healthier choices are being made with respect to milk purchases when the child was obese or perceived-to-be obese. This is particularly noteworthy given that narratives from mothers indicated a cultural preference for whole milk among Hispanic families, thus making it unlikely that these results would be due to a social desirability effect. A mother reported, *“*I used to drink whole milk because it's thick and has all the vitamins we need. Low-fat milk is watered down, looks like it isn't complete. Now I drink low-fat because at WIC they taught us the nutrients are the same, but whole milk has a lot more fat*.”* A WIC staff member also confirmed this cultural preference for whole milk in the following statement, “When I was in WIC, I used to think whole milk was better too because it means “complete” milk in Spanish. It means you get everything in there, and since you're paying for it, might as well get the whole milk then. That's what Hispanic families think. But with low-fat, you don't get everything because it's considered to be watered down.*”*

The obesity epidemic has produced a shift in body image in which the range for healthy weight is expanding to include those mildly overweight as the new normal [40]. While this may be a possible explanation for the dissonance, results from interview data suggested there may be a cultural preference, particularly among our Hispanic mothers and grandmothers, for a fuller size child (Figure 1). As one clinic staff reported, “Mothers do not think their kid is chubby, they think it's cute. While I was counseling one mother about her child's weight, she said his weight was fine and then called him, *“gordito”* (“little fat one,” a common term of affection).” These researchers reported similar cultural perceptions about children's weight; Hispanic mothers preferred a thin figure for themselves but a fuller figure for their children, as this is a sign of health and vitality [40–42]. Others found Hispanic mothers did not perceive their overweight child to be overweight, but rather “big boned,” “thick,” or “solid,” which is considered culturally acceptable and even desirable [43].

Our study also revealed a perception gap between the biomedical weight measure in children and how mothers understand and perceive child weight status. These findings are corroborated by other researchers, who also concluded that parents of young children had lower awareness of their child's weight status [44]. Findings from the narratives in Figure 1 illustrate that mothers defined healthy weight for their child as a function of (1) the child's ability to be as active as other children their age, regardless of their BMI percentiles; (2) the pediatrician's opinion of the child's weight status. As one mother shared, healthy weight is defined in the following way: “When they can run around and breathe properly, not out of breath. Kids who are chubby are out of breath all the time when they just walk.” These findings are supported by others who also found that mothers defined their child's weight not based on BMI percentiles, but more functionally, such that a child was “eating well and physically active” [43, 45].

In contrast, health practitioners such as WIC staff defined a child's weight status based on BMI percentiles. WIC paraprofessional staff confirmed these findings and recognized this disconnect as they counseled participants and lamented that because mothers did not recognize overweight in their child, they were not concerned about their child's weight (Figure 1). Chamberlin et al. reported that WIC staff in Kentucky also faced similar challenges in working with mothers who did not perceive the high BMI percentiles to be worrisome for their child [46]. WIC has great potential to make an impact in obesity prevention among preschool-aged children, but this disconnect may pose a problem in their efforts. If mothers do not perceive their child's weight as a problem, there will be less motivation to participate in prevention programs [47].

In addition, it is important to recognize the prominent role pediatricians have in alerting Hispanic parents to the problem of overweight in their child. Among Hispanics, physicians are held in a position of high authority, and parents usually follow their counsel and instructions. One mother shared, “When we go to the doctor, he decides if [my child] is at a healthy weight.” For mothers in our study, the doctor had the primary role in determining her child's weight status. Consequently, understanding and leveraging the important role doctors play in the Hispanic community by building partnerships between WIC and local pediatricians can move us toward bridging this perception gap to increase parental awareness of overweight. Knowledge sharing and communication between WIC staff and the pediatrician can create a supportive setting for parents to discuss their child's weight and strategies for adoption of healthy habits. In their meta-analysis of maternal underestimates of child's weight, Lundahl and colleagues found that half of parents underestimated their child's overweight/obese status; these researchers also suggested that pediatricians are well positioned to remedy parental underestimates by bringing awareness to parents and encouraging healthy habits, namely, diet and physical activity, early in life [48].

In 2009, changes to the WIC food packages changed allowable milk options for women and children older than 2 years of age such that whole milk could no longer be purchased with WIC benefits [49]. This study was conducted prior to the WIC package revisions, so we are confident that the food package restrictions were not what lead to reported feeding behavior of the reduced-fat milk.

While the findings support the literature on maternal perceptions of childhood obesity, this study was not without its limitations. As data were primarily from one major area in Los Angeles and focused primarily on Hispanics of Mexican descent, these findings may not be generalizable to other WIC programs around the US with different ethnic/racial compositions. Also, we utilized self-reported milk intake data, which limits our ability to quantify actual consumption levels. We also only focused on milk, which is a primary food in WIC, so these findings are only the starting point for understanding how perceptions may impact actual food choices. Despite these limitations, our mixed methods approach of combining two data sources and triangulating survey data with interviews from staff and participants provides a more complete and nuanced understanding of how maternal perception is associated with feeding behavior of milk in pre-school aged children.

## 5. Conclusion

Childhood obesity has reached epidemic proportions, and low-income Hispanic children remain particularly at risk. Results from this study expand upon existing work and demonstrate that maternal perceptions of self weight and child weight status are associated with her decisions regarding milk choice, namely, between reduced fat versus whole milk. Mothers in our study have some level of recognition of obesity in themselves and that of their child; as such, this was associated with mothers making healthier choice with regard to milk. Secondly, understanding the cultural context for how Mexican-American mothers perceive weight in themselves and their child is important for the development of obesity prevention strategies within this population. Finally, the role of health providers cannot be understated in this community. As such, future programming of WIC services for obesity prevention needs to be taken into consideration these cultural perceptions of weight among Hispanic participants.

## Figures and Tables

**Table 1:** Demographic characteristics of mother and child.

*Mother*		
Age in years (avg ± SD)	30.6 ± 5.5	
Years resided in the US (avg ± SD)	15.1 ± 6.8	

Birthplace	*n*	(%)
Born in the US	102	19.3
Foreign born	427	80.7

English language proficiency		
Very well or well	170	32.1
Not well	157	29.7
Not at all	202	38.2

Education level		
No formal schooling	4	0.8
8th grade or less	148	28
9–12th grade	187	35.3
High school graduate	115	21.8
Some college or graduate	75	14.2

Ethnicity		
Mexican	499	94.3
Salvadoran	16	3
Guatemalan	5	1
Puerto Rican	2	0.4
Other Hispanics	7	1.3

Weight status^*∗*^		
Underweight	1	0.2
Normal weight	106	20
Overweight	157	29.7
Obese	265	50.1

*Child*		
Age in years (avg ± SD)	3.9 ± 6.7	

Birthplace	*n*	(%)
Born in US	489	92.4
Foreign born	40	7.6

Gender		
Female	264	50
Male	265	50

Ethnicity		
Mexican	498	94.2
Salvadoran	15	2.8
Guatemalan	5	1
Puerto Rican	1	0.2
Other Hispanics	10	1.8

Weight status^*∗∗*^		
Normal weight	278	52.6
Obese	251	47.4

^*∗*^Mother's weight status based on CDC standards using BMI. ^*∗∗*^Child's weight status based on CDC age- and gender-specific BMI percentiles.

**Table 2:** Accuracy of maternal perception by weight status of self and child.

	Correct normal weight	Correct obese	Incorrect normal weight^*∗*^ (actually obese)
	*n* (%)	*n* (%)	*n* (%)
Mother's perception of her own weight status	104 (20%)	282 (53%)	140 (26%)
Mother's perception of child's weight status	277 (52%)	60 (11%)	191 (36%)

^*∗*^Mother's misperception: she perceives her child to be normal weight when the child is actually obese by anthropometric measurements.

**Table 3:** Actual and perceived weight status and milk intake.

	Reported milk intake % (*n*)			
	Whole	Reduced fat	Other^*∗∗∗*^	
Mother's actual weight^*∗*^				
Normal weight	44.2% (46)	51% (53)	4.8% (5)	*p* < 0.05^a^
Overweight/obese	30.3% (129)	66.7% (284)	3.1% (13)	

Mother's perceived weight				
Thin	25.8% (8)	74.2% (23)	0%	*p* < 0.05^b^
Normal	40.8% (89)	56% (122)	3.2% (7)	
Overweight	**28% (78)**	**68.1% (190)**	3.9% (11)	

Child's actual weight^*∗∗*^				
Normal weight	41.7% (123)	54.9% (162)	3.4% (10)	*p* < 0.01^c^
Obese	**28.2% (73)**	**68.7% (178)**	3.1% (8)	

Child's perceived weight				
Thin	41.3% (26)	52.4% (33)	6.3% (4)	*p* < 0.05^d^
Normal	36.9% (158)	60.5% (259)	2.6% (11)	
Overweight	**19.4% (12)**	**75.8% (47)**	4.8% (3)	

^*∗*^Mother's weight based on BMI. BMI < 18.5, thin; BMI between 18.5 and 24.99, normal; BMI ≥ 25, overweight; BMI ≥30, obese. ^*∗∗*^Child's weight based on CDC BMI for age growth charts. Normal weight is 5th percentile to less than the 85th percentile. Obese is equal to or above the 95^th^ percentile. ^*∗∗∗*^Other milks included soy, rice, and chocolate. Due to low frequency, these were grouped together. ^a^By using the *χ*^2^ test for percentages comparing maternal weight using CHIRP data. ^b^By using the *χ*^2^ test for percentages comparing mothers' perception of self-weight using CHIRP data. ^c^By using the *χ*^2^ test for percentages comparing normal and overweight children using CHIRP data. ^d^By using the *χ*^2^ test for percentages comparing maternal perception of child's weight using CHIRP data.

## Data Availability

The CHIRP data used to support the findings of this study are restricted by the CA Department of Public Health in order to protect WIC participants' privacy and confidentiality. Data are available from PHFE-WIC for researchers who meet the criteria for access to confidential data.

## Additional Points

*Significance*. This study documents that, despite maternal misclassification of self-weight and child's weight, mothers do seem to be making healthy feeding choices if the child was obese. Cultural understanding of healthy weight is nuanced and does not necessarily track with the biomedical BMI categorization; therefore, understanding how Hispanic mothers define healthy weight is vital for the development of prevention programs. Pediatricians serve as vital connecting points in obesity prevention efforts in Hispanic communities.
